# A dataset on quantifying a mass mortality event in freshwater wildlife within the Lower Odra River

**DOI:** 10.1016/j.dib.2023.109753

**Published:** 2023-11-11

**Authors:** Dominik Marchowski, Agnieszka Szlauer-Łukaszewska, Łukasz Ławicki, Jacek Engel, Ewa Drewniak, Karol Ciężak

**Affiliations:** a Ornithological Station, Museum and Institute of Zoology of the Polish Academy of Sciences, Poland; b Institute of Marine and Environmental Sciences, University of Szczecin, Szczecin, Poland; c Eco-Expert, Szczecin, Poland; d Greenmind Foundation, Warszawa, Poland; e Save the Rivers Coalition, Poland; f Naturalist Club, Owczary, Poland; g The Society for Earth, Oświęcim, Poland

**Keywords:** Bivalvia, Gastropoda, Fish, Golden Algae, Ecosystem collapse, Ecosystem Imbalance, River pollution, Ecological disaster

## Abstract

In response to the significant ecological disaster in the Odra River during the summer of 2022, a comprehensive data collection process was initiated to quantify the extent of mortality among aquatic species. The dataset focuses on the downstream section of the river, identified as the area with the highest accumulation of deceased organisms. The data collection involved systematic sampling and counting of dead organisms, including fish, bivalves , and aquatic snails. Special attention was given to specific species such as Unionidae mussels, *Anodonta anatina, Sinanodonta woodiana*, and *Viviparus viviparus*. Additionally, transects were designated for focused data collection on fish mortality. The dataset provides detailed mortality figures, biomass estimates, and percentage reductions for each species. This comprehensive dataset holds significant potential for reuse by researchers studying the effects of toxins on freshwater ecosystems, the impact of invasive species on native populations, and conservationists aiming to restore the affected areas.

Specifications Table

**Table:** 

Subject	Wildlife, Bivalvia, Gastropoda, Fish, Prymnesium parvum, Golden Algae, Invasive species
Specific subject area	Description and documentation of the ecological disaster on the river, Ecosystem collapse, Ecosystem Imbalance, River pollution, Ecological disaster, Mass mortality, River ecosystem
Data format	Raw data tables in text format, prepared for analysis using the R programming environment.
Type of data	Raw table, R code
Data collection	i. Qualitative studies on observation points, binary effect determination, and identification of taxonomic groups if present. ii. Benthic sampling conducted in 2017 and 2022. iii. Counting dead mussels along transects in the riparian zone of the river. iv. Counting dead fish along transects in the riparian zone of the river. v. Counting dead mussels flowing down the river from an observation point.
Data source location	Country: Poland and Germany, border river Odra. i. Starting point of the ecological disaster: 50.911056, 17.410776 (Lipki, Poland), ecological disaster endpoint: 53.434170, 14.576252 (Szczecin, Poland), midpoint of accumulation of ecological disaster - main research area, lower Oder: 52.973591, 14.169900 (Odra border Poland - Germany).
Data accessibility	Repository name: Mendeley Data Data identification number: 10.17632/985dh5r9cs.1 Direct URL to data: https://doi.org/10.17632/985dh5r9cs.1
Related research article	Szlauer-Łukaszewska, A., Ławicki, Ł., Engel, J., Drewniak E., Ciężak K., Marchowski D. 2023. Quantifying a mass mortality event in freshwater wildlife within the Lower Odra River: Insights from a large European river. Science of the Total Environment 167898 https://doi.org/10.1016/j.scitotenv.2023.167898

## 1 Value of the Data

• The data provides a comprehensive account of the ecological disaster's impact on the River Odra in 2022, detailing the mass mortality rates among various aquatic species, including fish, bivalves, and water snails.
• This dataset offers a unique insight into the effects of toxins released by the haptophyte golden algae, *Prymnesium parvum*, on freshwater ecosystems, serving as a reference for future studies on similar ecological disturbances.
• With detailed mortality statistics for species such as Unionidae mussels, *Anodonta anatina, Sinanodonta woodiana, Unio pictorum*, and *Unio tumidus*, researchers and policymakers can better understand the disaster's magnitude and its implications for the river's biodiversity.
• The data on fish mortality, spanning across a 150 km stretch of the lower Odra, provides a clear picture of the disaster's impact on the river's ichthyofauna, aiding in conservation and recovery efforts.
• This dataset is invaluable for environmental scientists, conservationists, policymakers, and local stakeholders, offering a foundation for devising strategies to restore and rehabilitate the affected river sections.
• The data can be reused in future studies to monitor the river's recovery progress, assess the long-term impacts of such ecological disasters, and develop preventive measures against potential future occurrences.
• By understanding the extent of the disaster and its effects on various species, regulators and local authorities can formulate more effective strategies to mitigate the impacts of similar events in the future, ensuring the preservation of freshwater ecosystems.

## 2 Background

The Odra River, representative of temperate lowland rivers in Central Europe, is renowned for its biodiversity, particularly in the Lower Odra Valley section. [2]. Historically celebrated for its aquatic richness, it supports diverse fish species and other aquatic creatures, including the Eurasian beaver (*Castor fiber*) and the Eurasian otter (*Lutra lutra*) [3,^4^,5]. Its invertebrate population plays a crucial role in maintaining ecosystem health [6,7]. However, the 2022 ecological disaster profoundly affected this vibrant ecosystem [8]. This article, an extension of a prior publication [1], aims to assess the disaster's impact, particularly in the Odra's lower section, which bore the brunt of the catastrophe. The study focuses on the mortality of three primary wildlife groups: mussels, water snails, and fish. It evaluates the response of individual mussel species from the Unionidae family, quantifies the mortality of Bivalvia, and offers a comprehensive assessment of fish mortality. By understanding these dynamics, this research provides insights for future conservation and rehabilitation efforts.

## 3 Data Description

**Fig. 1:**
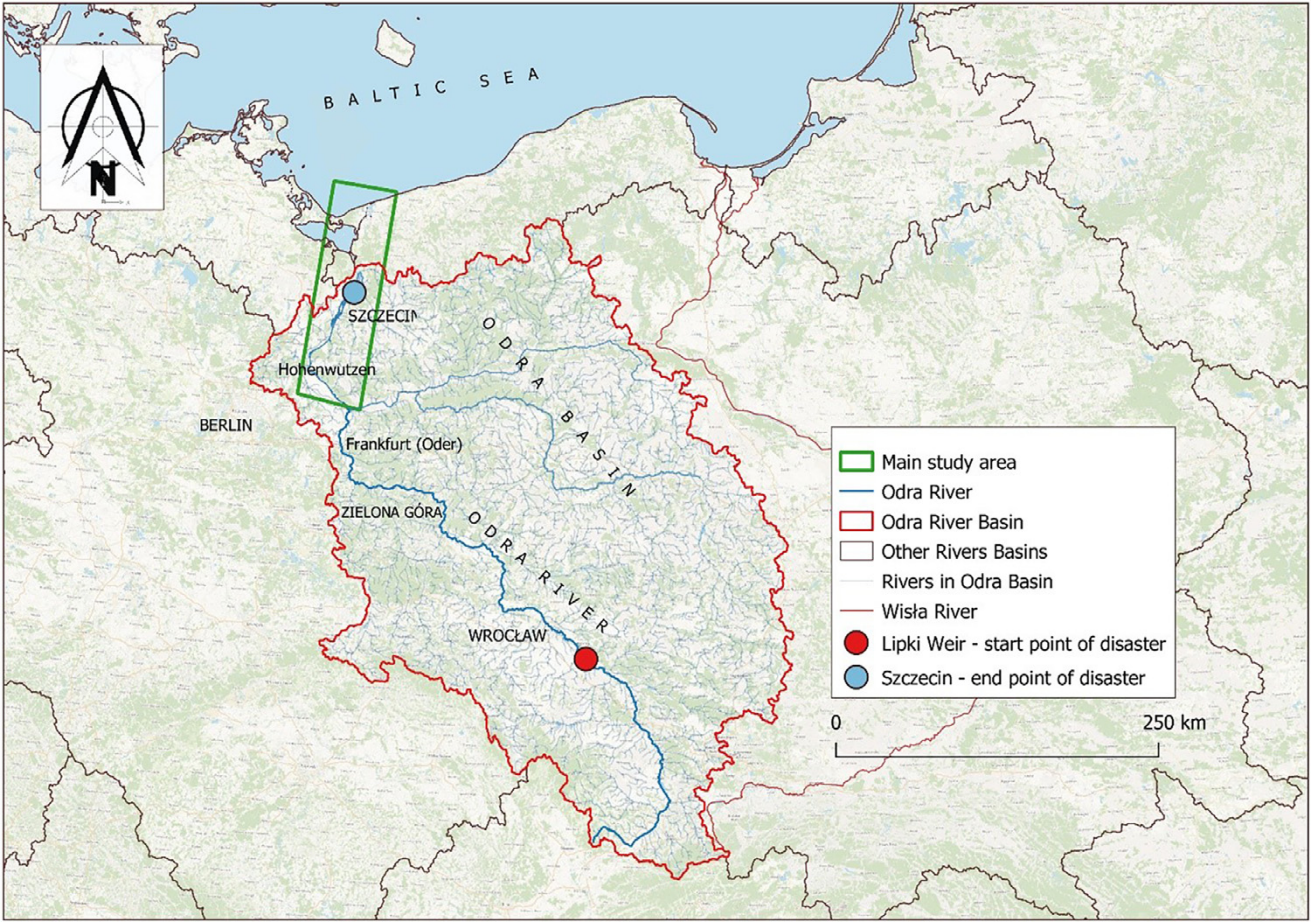
Map of the Odra River basin, highlighting the start and end points of the 2022 ecological disaster and delineating the study area (source: Fig. 3 from Szlauer- Łukaszewska et al. 2023 [1]).

In this study, we focus on the ecological disaster that unfolded along the Odra River, a border river traversing both Poland and Germany [8]. The ecological disaster's inception point was in Lipki, Poland, at coordinates 50.911056, 17.410776 and culminated in Szczecin, Poland, at coordinates 53.434170, 14.576252. The epicenter of our research, representing the midpoint of the disaster's accumulation, is situated in the lower Odra, near the Poland-Germany border, at coordinates 52.973591, 14.169900. A detailed outline of the research area is depicted in Fig. 1. The disaster spanned from July 26, 2022, marked by initial reports of fish fatalities in Poland, until August 30, 2022. By the end of this period, while remnants of dead fish and mollusks were still visible in the lower Odra, there were no indications of further deaths [9].

### 3.1 Benthos samples – mussels and snails

The first dataset pertains to benthic sample collections conducted at 13 consistent sampling points in the years 2017 and 2022 (Table 1). These samples were taken from various locations, including Bielinek, Cedynia, Kaleńsko, Kostrzyn nad Odrą, Kunice, Ługi Górzyckie, Ognica, Pamięcin, Piasek, Pławidło, Porzecze, Stara Rudnica, and Słubice. The taxa covered in the dataset include *Anodonta anatina, Sinanodonta woodiana, Unio pictorum*, and *Unio tumidus*. The size column indicates the number of individuals of the specimens collected, with some entries having a value of zero, indicating no specimens of that particular taxa were found at that location during the sampling.

**Table 1:** Sampling points and dates for examining the number and density of mussels and water snails in 2017 and in 2022.

Point name	Coordinates	Date in 2017	Date in 2022
Kunice	52.263651, 14.650922	11.09.2017	5.11.2022
Pławidło	52.437683, 14.561907	11.09.2017	5.11.2022
Pamięcin	52.437661, 14.561824	11.09.2017	5.11.2022
Ługi Górzyckie	52.532913, 14.606456	11.09.2017	5.11.2022
Kostrzyn nad Odrą	52.582669, 14.621549	12.09.2017	6.11.2022
Kaleńsko	52.633846, 14.539146	12.09.2017	6.11.2022
Porzecze	52.669977, 14.461437	12.09.2017	6.11.2022
Cedynia	52.870383, 14.155140	12.09.2017	6.11.2022
Bielinek	52.945551, 14.145225	12.09.2017	6.11.2022
Piasek	52.989121, 14.222311	13.09.2017	7.11.2022
Ognica	53.059961, 14.354204	13.09.2017	7.11.2022
Stara Rudnica	52.821025, 14.188331	13.09.2017	7.11.2022
Słubice	52.337923, 14.564294	13.09.2017	7.11.2022

The study comprises three distinct datasets, each tailored for different types of analyses. While they all share common columns such as date, research point (station), species (taxa), and number of individuals (size), each dataset has its unique attributes:

(a) mussels_2017-2022_2.txt: This dataset includes a “freq” column. It uses a zero-to-one method to indicate the frequency of occurrence of a particular species at a specific research point.
(b) mussels_2017-2022_3.txt: This dataset features a “freq_all” column. It denotes whether at least one individual from any mussel species was recorded at a given research station.
(c) mussels_data_2017-2022_4.txt: This dataset introduces a “method” column, which specifies the sampling method employed during data collection. Two methods were employed for the collection: ‘driver’ and ‘net’.

The dataset “percentage_2017-2022.txt” provides an overview of the average percentage distribution of various mussel species across all 13 research points in the years 2017 and 2022. The data is summarized as follows:

*Anodonta anatina* (AA): Represented 30 % of the mussels in 2017 and decreased to 11.2 % in 2022.

*Sinanodonta woodiana* (SW): Made up a mere 0.812 % in 2017 but saw an increase to 5.59 % in 2022.

*Unio pictorum* (UP): Accounted for 37.3 % in 2017 and rose significantly to 63.5 % in 2022.

*Unio tumidus* (UT): Constituted 31.9 % in 2017 but dropped to 19.7 % in 2022.

The dataset “Water_snalis_2017-2022.txt” presented here pertains to the Viviparidae family and is derived from the same 13 research points that were used for the mussel study (Table 1).

### 3.2 Dead mussels transect count data

The dataset “Musssels_GEE” presented pertains to mussel counts conducted along transects adjacent to a riverbank. The river's kilometer markers have been uniquely encoded to facilitate the use of the “rtrim” package in R. This package, typically employed for calculating population growth rates within specific time windows, has been innovatively repurposed in this study to determine the growth rate along the river's course. For each year, a corresponding river kilometer value has been assigned.

From 623.7 to 733.2 km of the river, the data showcases the mussel counts (“count”) at specific river kilometer markers (“km”) for site “101”. It's evident that there are several river kilometer markers where the mussel count is not available (denoted as “NA”).

The “rtrim” package is particularly adept at predicting unknown values, making it a valuable tool for estimating mussel counts at these unspecified river kilometer markers. Given the limited applicability of the rtrim package for assessing population trends within specific time intervals, a novel approach was adopted. By artificially attributing river kilometers to the representative time interval (1993-2018), the average growth rate along the river was computed. It's crucial to understand that this methodology is a technical adaptation. Here, the 'year' variable essentially serves as a proxy for the encoded 'km' variable, providing insights into the spatial distribution and abundance of mussels along the river.

### 3.3 Dead mussels flowing in the river current count from observation point

The dataset “point_observations_2022.txt” provides a snapshot of observations made on August 17, 2022, from a vantage point situated on the riverbank. The primary objective of these observations was to count the number of dead mussels flowing with the river's current.

Each row in the dataset represents a separate observation session, with the following details: date: The date of observation, which is consistently August 17, 2022, for all entries. id: A unique identifier for each observation session. In this dataset, there are five sessions labeled from S1 to S5. size: The count of dead mussels observed during the session. For instance, during the session with ID “S1”, 23 dead mussels were counted. time: The duration of each observation session in minutes. All sessions in this dataset lasted for 5 minutes.

From this data, it can be inferred that during these five sessions, a total of 77 dead mussels were observed flowing in the river over a combined observation time of 25 minutes.

### 3.4 Counting dead snails in photos

This dataset “snails_count_2022.txt” provides a detailed account of the number of snails and mussels observed in photographs taken along the riverbanks during a disaster event. The data was collected on specific dates in August 2022 at various sites along the river.

Columns Explanation:

pic: Refers to the name or identifier of the photograph.

snails: Represents the count of snails observed in the respective photograph.

mussels: Represents the count of mussels observed in the respective photograph.

date: The date on which the photograph was taken.

site: The specific location or site where the photograph was captured.

N: Latitude coordinate of the site.

E: Longitude coordinate of the site.

The dataset also includes summary statistics:

Total: The total count of snails and mussels across all photographs.

Mean: The average count of snails and mussels per photograph.

sd: Standard deviation, indicating the variability in the counts across photographs.

pier: Square root of the variance, providing another measure of spread.

se: Standard error of the mean, indicating the precision of the sample mean estimate.

95 %CI+: The upper limit of the 95 % confidence interval for the mean.

95 %CI-: The lower limit of the 95 % confidence interval for the mean.

This dataset offers a comprehensive view of the impact of the disaster on the snail and mussel populations in the river, as observed from the riverbanks. The photographs serve as a visual record, while the counts and statistical measures provide a quantitative assessment of the situation.

### 3.5 R code – snails count

This R code provides a series of calculations and analyses based on the data of snails and mussels observed in photographs taken along the riverbanks during a disaster event. Here's a concise description of the code:

*Data Creation:*

A dataframe named data is created containing information about the pictures, counts of snails and mussels, date of the photograph, and the site where the photograph was taken. The latitude (N) and longitude (E) columns are initialized but not filled with data in this snippet.

*Basic Statistical Calculations:*

The total counts of snails and mussels are calculated.

The mean (average) counts of snails and mussels are computed.

The standard error (SE) for both snails and mussels is calculated.

95 % confidence intervals (CI) for the mean counts of snails and mussels are determined.

*Display Results:*

The calculated values (total, mean, SE, and 95 % CI) for both snails and mussels are printed to the console.

*Estimation of Snail Population:*

An estimated total count of mussels is provided.

The proportion between the observed counts of snails and mussels is calculated.

Using this proportion, an estimated count of snails is determined based on the provided total count of mussels.

*Confidence Interval Calculations for Estimated Snail Population:*

Given mean counts and 95 % CI for snails and a total count and 95 % CI for mussels, the 95 % CI for the estimated snail population is calculated.

The code essentially provides a comprehensive statistical analysis of the snail and mussel populations based on the observed counts in the photographs. It also offers an estimation of the snail population using the observed proportion between snails and mussels.

### 3.6 Dead fish count

This dataset “Fish_2022_Odra_Disaster_km_river.txt” provides detailed information about dead fish collected during a disaster event. Here's a breakdown of the dataset:

Date: The date when the observations were made.
Site: A descriptive name for the location where the observations were made.
Site_ID: A unique identifier for the observation site.
Shore_habitat: Describes the type of habitat on the riverbank where the observations were made (e.g., Gravel).
Species: The scientific name of the fish species observed.
Spec_PL: The common name of the fish species in Polish.
Size: The number of individuals of the dead fish observed.
Animal_group: The broader classification of the observed species (in this case, all are classified as “Fish”).
dead-alive: Indicates whether the fish was found dead (1) or alive (NA). It seems that '1′ indicates dead fish, while 'NA' might indicate that the status was not recorded.
SN: A serial number or identifier for the observation.
Section: A section or segment of the observation area.
km: The kilometer marker or specific location along the river where the observation was made.
From the provided data:
Observations were made at different sites, such as “Zaton_Dolna_8”.
Various fish species were observed, both by their scientific names (e.g., “Ctenopharyngodon_idella”) and their Polish common names (e.g., “Amur”).
The dataset provides counts of each species observed at each site on specific dates.
The “Shore_habitat” column indicates that these observations were made in gravel habitats along the riverbank.
The dataset also provides specific kilometer markers, indicating the exact location along the river where each observation was made.

In summary, this dataset offers a comprehensive record of dead fish observed at specific sites along a river following a disaster event. The data includes details about the species, count, location, and date of observation.

## 4 Experimental Design, Material and Methods

This section details the analytical methods applied to the databases discussed in this paper. The methodology for the overarching research, which is the focus of the primary paper, is available in reference [1]. Comprehensive details, including all codes compatible with the databases, are provided in a dedicated methodological paper [10].

### 4.1 Benthos samples – mussels and snails

Mussels and water snails were gathered from 13 specific sites (Table 1) from September to November in 2017 and 2022 using a uniform approach. This method combined diving and net sampling techniques to average results.

In the diving technique, a diver collected mussels directly from the riverbed. Simultaneously, a specialized net with a hydrobiological mesh was used to dradge the riverbed, capturing both mussels and snails. This mesh, 25 cm wide with a 405 µm mesh size, covered a 0.625 m² area during each sampling, standardizing densities to 1 m². In total, an area of about 50 m² was examined in both years.

Average density values were calculated for all sites for each year. We then determined the percentage representation of each taxon and their frequency across sites.

Data normality was checked using histograms, Q-Q plots, and the Shapiro-Wilk test. Depending on the data's distribution, we either used a one-way ANOVA (for normally distributed data) or the Kruskal-Wallis test (for non-normal data) to compare yearly results. The goal was to identify significant year-to-year variations in sample parameters.

### 4.2 Dead mussels transect count data

Following the ecological event on the Odra River, we conducted a survey to count the dead mussels that had washed up on the shores. We selected twelve ten-meter sections at random in the central lower Odra River, covering an 18-kilometer stretch from Zatoń Dolna to Widuchowa. Two additional sections were added at the start and end of this study area. Our analysis focused on the main river course, a 109 km segment from Kaleńsko to Szczecin, with the observed area representing 16.5 % of this distance.

We first examined how the mussel count related to the site and the river's kilometer marker. Using generalized additive modeling (GAM), we assessed the link between mussel numbers and these factors, considering potential non-linear associations [11]. We then used the Generalized Estimating Equations model (GEE) to determine how mussel numbers changed across different river sections. This model also helped predict mussel counts for the entire lower Odra River, from Kaleńsko to Szczecin [12]. We averaged the model's predictions and expanded them over the entire river segment. The formula used was:

$$\bar{x}=\frac{1}{n}\sum_{i=1}^{n}x_{i}$$

Where *x1, x2,...xn* are mussel counts from the 10 m sections and predicted counts for subsequent sections.

$$\hat{x}=2L\bar{x}$$

Here, $\hat{x}$ is the extrapolated result, and *L* is the river's length. We also included the 26 km Western Odra section, using the average predicted mussel count for the Widuchowa – Szczecin segment.

### 4.3 Dead mussels flowing in the river current count from observation point

During the ecological crisis on the Odra River, we counted dead mussels from the Unionidae family seen floating in the river. This count was based on the point transect method [13]. On August 17, 2022, in Krajnik Dolny, we conducted five five-minute counting sessions at two-hour gaps. During these, we recorded all dead Unionidae mussels floating by.

The procedure involved watching the mussels as they floated past a set line perpendicular to the riverbank, aligning with the strip transect sampling approach [14]. We then adjusted the data for visibility, using a 0.6 detection rate from Ronconi and Burger [15]. The average count per minute was scaled up to estimate the daily count, factoring in the detection rate.

$$\bar{x}=\left[\left(\frac{1}{n}\sum_{i=1}^{n}x_{i}\right)/\omega \right]/5$$

Where x is the count in the sample, n is the sample size, and ω is the detection rate.

This average was multiplied by 60 minutes and then by 24 hours to estimate the daily flow of mussels. We also calculated 95 % confidence intervals for this value.

Considering the river's flow speed, we estimated how far the mussels might have traveled before reaching our counting spot. Data from the State Water Holding Polish Waters (found at https://www.pgw.wody.gov.pl) helped us gauge the river's average flow, allowing us to estimate the mussels' likely starting point.

### 4.4 Counting dead snails in photos

In the Lower Odra Valley section, extending from Kostrzyn to Szczecin, sporadic images of the river's banks were taken to record the various animal species found lifeless and stranded. Most of these animals primarily fell into three categories: fish, mussels, and aquatic snails.

**Fig. 2:**
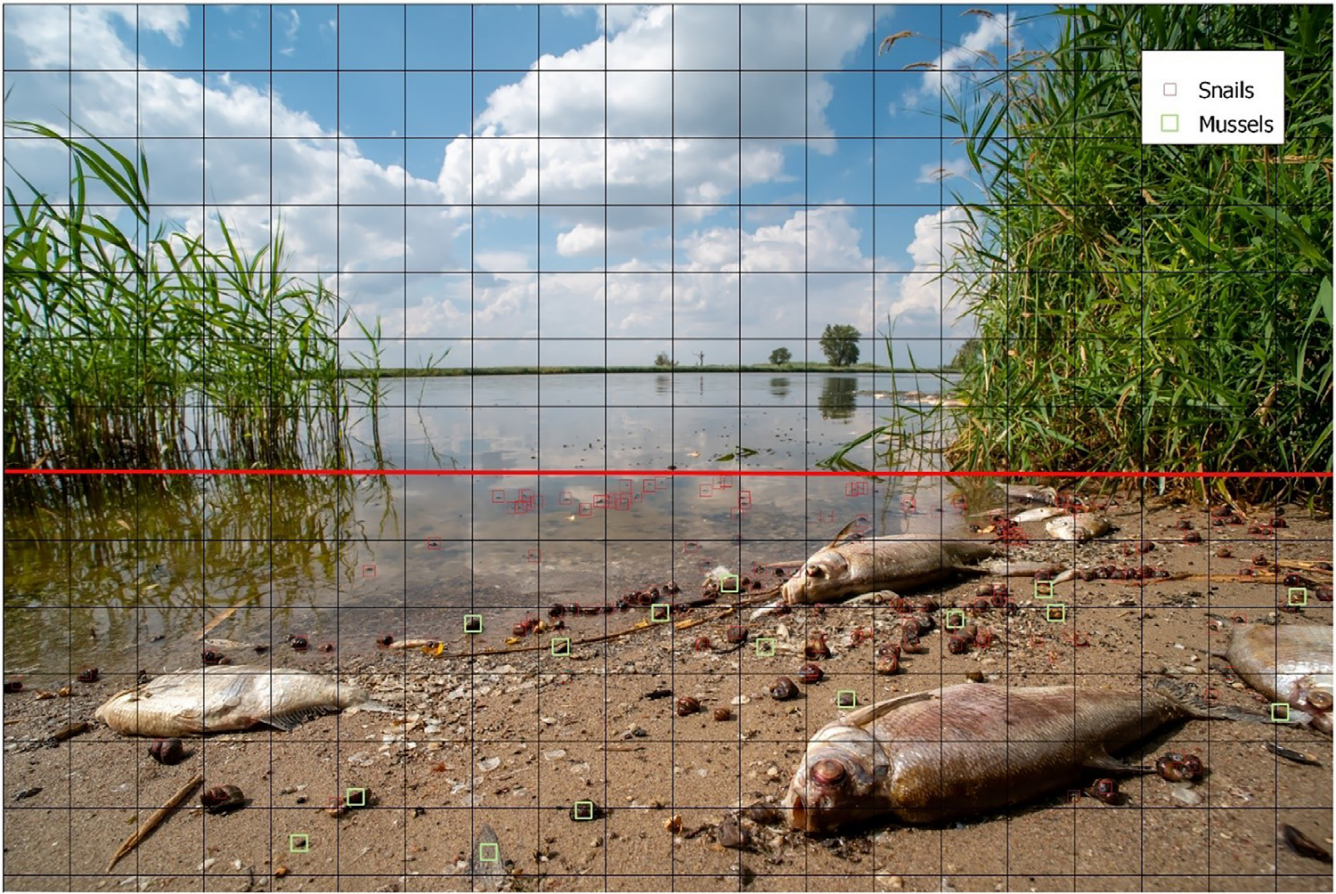
An exemplary annotated photo on the basis of which the proportion of dead snails in relation to mussels was assessed. Red demarcation line – objects beyond this line were not included in the count.

**Fig. 3:**
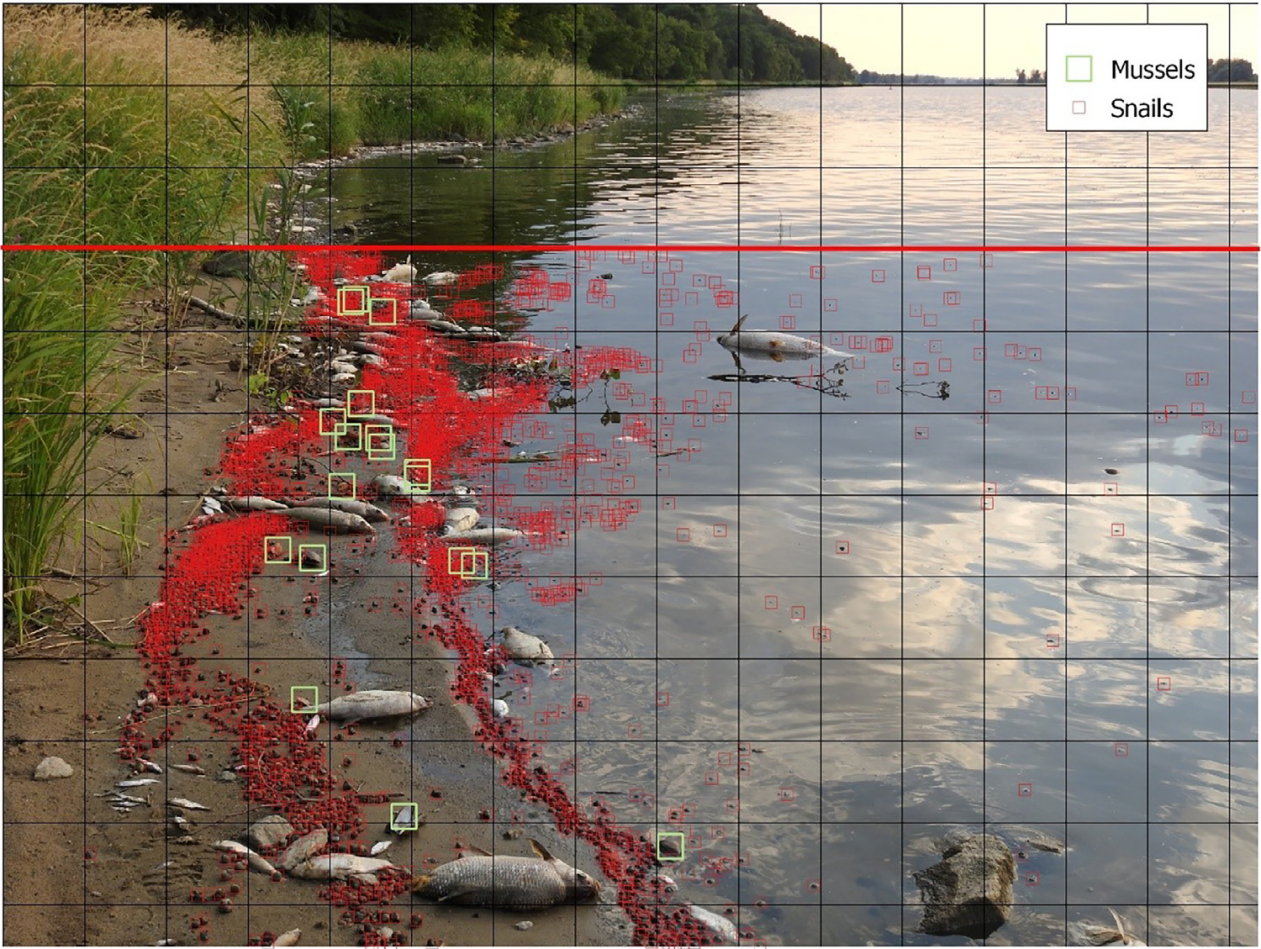
An exemplary annotated photo on the basis of which the proportion of dead snails in relation to mussels was assessed. Red demarcation line – objects beyond this line were not included in the count.

For both in-depth and broad analyses, these photos (N=13) were marked similarly to the methods used for preparing data for machine learning neural networks [16,17]. A square grid was superimposed onto each photo to aid in counting. Photos captured with perspective were examined to ensure proper object recognition. In cases where recognition became inadequate, a demarcation line was drawn. Objects beyond this line were not included in the count (Fig. 2, Fig. 3).

By contrasting the average snail count with the mussel count and considering the projected number of mussels found ashore we deduced the probable count of deceased aquatic snails through proportional analysis.

### 4.5 R code – snails count

The provided R code is designed to analyze data related to snails and mussels obtained from photographs. Here's a brief overview of its methodology.

Data Initialization: The code begins by creating a dataframe named 'data' that contains information from various photographs. This dataframe captures details like the picture name, counts of snails and mussels, the date the photo was taken, the site of the photo, and coordinates (N and E) which are initially set as NA (Not Available).

Basic Calculations: The code then calculates the total number of snails and mussels across all photographs. It also computes the mean for both snails and mussels.

Statistical Analysis: The standard error (SE) for both snails and mussels is calculated. This is followed by the determination of the 95 % confidence intervals (CI) for the mean values of snails and mussels. The CI provides a range within which the true population mean is likely to fall 95 % of the time.

Estimation Based on Proportions: An estimated number of mussels is provided, and the proportion of snails to mussels is calculated based on the previously computed totals. Using this proportion, the estimated number of snails is determined.

Display Results: The results of the above calculations, including total counts, means, SEs, and CIs, are printed to the console for easy interpretation.

Further Calculations: The code then provides specific mean numbers and CIs for snails and mussels. Following this, it calculates the CI intervals for snails based on the given mussels' CI.

### 4.6 Dead fish count

In the heart of the Lower Odra Valley, we chose 43 transects, each 10 meters long, spread over a 20 km stretch from Zatoń Dolna to Widuchowa. These transects covered various habitats along the river, such as stony, gravelly, reed-covered, and sandy banks.

We meticulously counted all typical vertebrates within these transects, focusing on species living in or near water that might be impacted by the disaster. This encompassed creatures like lampreys, fish, amphibians, birds, and mammals. While fish underwent a detailed quantitative analysis, other animals were qualitatively assessed [1].

For fish counts, we used a similar extrapolation method as for mussels. The final count was then translated into weight to align with standard reporting formats in other studies. We derived data on fish sizes from two sampling points in the Odra's estuary section in Szczecin, where 4,508 dead fish from 24 affected species were measured [18]. To determine fish weight, we used average lengths and a recognized conversion tool with species-specific factors from a known website.

Statistically, we applied the Kruskal-Wallis test and the Generalized Additive Model (GAM) to study the link between fish numbers and their locations. The former tested for variations in fish counts across sites, while the latter analyzed the nuanced relationship between fish counts and location, factoring in other variables.

## Limitation

While the dataset provides a comprehensive overview of the ecological disaster's impact on the Odra River's aquatic life, there are inherent limitations to consider. Firstly, the data collection was constrained by the vastness of the affected area, potentially leading to under-sampling in certain regions. This might result in an underestimation of the total mortality figures for some species. Secondly, due to the rapid decomposition of some organisms, especially in warmer conditions, there's a possibility that not all deceased specimens were accounted for during the sampling process. Additionally, biases might have been introduced during data collection, as areas with higher visibility and accessibility were more frequently sampled than remote or obscured sections. Lastly, while efforts were made to ensure accurate species identification, there remains a margin of error, particularly with juvenile or damaged specimens. It's essential to interpret the dataset with these limitations in mind.

## Ethics Statement

The authors adhere to the ethical guidelines of the journal. While this research involved activities with wild animals, all procedures were in strict compliance with national law and European Union regulations. No humans or data from social media were involved in this study.

## CRediT authorship contribution statement

**Dominik Marchowski:** Conceptualization, Data curation, Formal analysis, Funding acquisition, Investigation, Methodology, Project administration, Supervision, Validation, Visualization, Writing – original draft, Writing – review & editing. **Agnieszka Szlauer-Łukaszewska:** Conceptualization, Data curation, Formal analysis, Funding acquisition, Investigation, Methodology, Project administration, Validation, Visualization, Writing – original draft, Writing – review & editing. **Łukasz Ławicki:** Conceptualization, Data curation, Investigation, Methodology, Validation, Writing – review & editing. **Jacek Engel:** Conceptualization, Data curation, Investigation, Methodology, Validation, Writing – review & editing. **Ewa Drewniak:** Conceptualization, Data curation, Investigation, Methodology, Validation, Writing – review & editing. **Karol Ciężak:** Conceptualization, Data curation, Investigation, Methodology, Validation, Writing – review & editing.

## Data Availability

Quantifying a mass mortality event in freshwater wildlife within the Lower Odra River: Data (Original data) (Mendeley Data)
